# Bilateral muscle compensation occurs with a unilateral rotator cuff tear: A modeling study

**DOI:** 10.1371/journal.pone.0333103

**Published:** 2025-09-29

**Authors:** Zoe M. Moore, Joshua Pataky, Meghan E. Vidt

**Affiliations:** 1 Biomedical Engineering, Pennsylvania State University, University Park, Pennsylvania, United States of America; 2 Physical Medicine and Rehabilitation, Penn State College of Medicine, Hershey, Pennsylvania, United States of America; Carol Davila University of Medicine and Pharmacy: Universitatea de Medicina si Farmacie Carol Davila din Bucuresti, ROMANIA

## Abstract

Bimanual tasks are necessary for daily activities but become difficult to complete with a musculoskeletal disorder. Rotator cuff tears (RCT) are a common musculoskeletal disorder. It is unclear how an unaffected contralateral limb compensates when performing bimanual tasks. This work used a computational model to examine the effects of a unilateral RCT on performance of loaded bimanual tasks. A bilateral upper limb model was developed in OpenSim (v3.3), then modified to represent 4 RCT severities ranging from no RCT to massive RCT. A shared load (13.3 N or 44.5 N) was added to the models to represent holding typical household and occupational objects. Three motions were used as inputs to the Computed Muscle Control algorithm: static postures at low and high arm positions, and a dynamic forward reach task. The point analysis tool was used to track the shared load position. Outcomes included muscle force normalized by peak isometric force and maximum hand position deviation relative to input kinematics. Quantitative trends were analyzed for each outcome. During static tasks, uninjured muscles on the injured side increased force contribution with increased external load and RCT severity. For the dynamic task, infraspinatus muscle force increased with the massive RCT severity on the unaffected side by 76% for the 13.3 N load and 91% for the 44.5 N load, indicating contralateral muscle compensation. Minimal deviation occurred for the position of the shared load during the low posture static and dynamic tasks. For the high posture static task there was an inferior (342 mm) position change toward the unaffected side (83 mm) with the massive RCT, 44.5 N load. The bilateral model developed here has provided insights into bilateral compensation with a unilateral RCT. This modeling tool can be used to enhance understanding of task performance in the context of rehabilitation.

## Introduction

The World Health Organization states that 1.71 billion people experience musculoskeletal pain worldwide. Shoulder disorders and injuries are estimated to account for 33% of all reported musculoskeletal pain, resulting in 570 million people affected [1]. Individuals who work in occupations that require repetitive load bearing are at greater risk for shoulder pain, as prior work shows that physically demanding occupations have an increased prevalence of shoulder injury compared to those who work in more sedentary occupations [2,3]. Many factors are related to the increased injury prevalence in the workplace, such as prolonged heavy lifting, overhead working, repetitive load bearing, and working in awkward positions [4]. A prior study reports that complaints of shoulder pain occur more frequently in those completing overhead work than those who do not [5]. Shoulder disorders due to strenuous working conditions can range from shoulder instability to large full-thickness tears of the rotator cuff tendons [4].

Rotator cuff tears (RCT) are a common musculotendon injury that can lead to increased pain and functional deficits [6]. The rotator cuff is comprised of the tendons from the supraspinatus, infraspinatus, subscapularis, and teres minor muscles. Rotator cuff tears are characterized by partial- or full-thickness tears of the supraspinatus tendon and are often accompanied by tears of the infraspinatus and subscapularis tendons [7]. A study reports that larger tear severities result in increased difficulty completing activities of daily living [8]. Many upper extremity tasks of daily living, both functional and occupational, require the use of both hands to be efficiently completed [5,9]. Despite bimanual tasks accounting for a large majority of daily functional tasks, prior work does not assess the effects of RCT on bilateral muscle compensation. Prior work does suggest that unilateral muscle compensation occurs in the context of RCT [10–12], but additional studies need to better understand the effects of a unilateral RCT on bilateral muscle compensation. Current treatments for RCT aim to restore function and range of motion [1], often focusing on only the injured side, but rehabilitation of the uninjured side may need to be considered during treatment to improve outcomes and a patient’s ability to return to work and daily activities. In order to inform clinical practices on rehabilitation, we must first understand how individual muscle forces are changing bilaterally in the context of unilateral RCT. This will improve our understanding of the muscle compensation that occurs with RCT, as prior work shows that unilateral muscle compensation increases with increasing RCT severity [12].

It is challenging or not feasible to experimentally measure some variables *in vivo*, such as individual muscle forces. Current methods to assess muscle activation include electromyography (EMG), which is not a direct measure of individual muscle force, and strength measurements, but it is not feasible to measure the strength of an individual muscle. Computational modeling allows for the study of predicted muscle force contributions from individual muscles during task performance. In addition, computational models allow us to examine the combined and isolated effects of factors, such as RCT severity, task, and external load, which can be difficult to account for in experimental study designs. However, computational models allow prediction of individual muscle forces while considering influential factors, including RCT severity and age, while also changing variables, like external load and task. The majority of upper extremity models that are available are unilateral models [13] or full body models that are primarily used for lower limb locomotion analysis [14]. One bilateral upper extremity musculoskeletal model is available, although verification of model kinematics was not reported [15]. The lack of validated bilateral models makes it difficult to examine the effect of bimanual tasks. Understanding the predicted changes and compensations that can occur during the performance of bimanual tasks will help guide future development of experimental studies to further evaluate occupational and functional task performance and design more targeted rehabilitation strategies. Therefore, the objective of this work was to further develop an existing unilateral model to obtain the contralateral side to examine the effects of a unilateral RCT on muscle compensation strategies while moving an external load for static and dynamic tasks. A bilateral model was developed in OpenSim Software (v.3.3) [16] and then modified to represent unilateral RCT of varying severities. This model was then used to predict the individual forces of muscles crossing the shoulder during performance of 3 tasks while holding a shared load. The tasks that were used are representative of functional tasks of daily living and occupational tasks [5,17]. A static hold at waist height was selected to represent holding or carrying a box at a fixed height. A static posture above eye height was selected to represent working overhead with no changes to shoulder degrees of freedom. A dynamic forward reach was selected to represent moving a shared load forward in the sagittal plane, representative of both occupational and common daily activities. It was hypothesized that muscle compensation would occur in uninjured muscles on the injured side and also in muscles on the unaffected side to account for the reduced muscle force from a unilateral RCT.

## Methods

### Model development

A bilateral model was developed by modifying the existing unilateral upper extremity model in OpenSim Software (v.3.3) [13,16]. This was accomplished by first creating the bone files for the contralateral side by reflecting the vertices in the native bone geometry files, then re-normalizing the coordinate system to reflect the bone from the right to the left side. The existing rotational definitions of the published and validated unilateral MoBL-ARMs [13] model were then mirrored in the local x- and y-directions (rotations about the z-axis) and the current translational definitions in the direction of the local z-axis (medial/lateral plane) to the contralateral side. This process was completed for all degrees of freedom, including the phantom degrees of freedom, in the model. Additionally, the center of mass positions of upper limb bodies were mirrored and each muscle path was added to the contralateral side.

### Kinematic verification

Following development, model kinematics were verified to ensure bilateral symmetry using two 7 s tasks: a static task in which the arm was by the side at 0 ° shoulder elevation, 0 ° elbow flexion, and the palm facing toward the midline at −25 ° shoulder rotation; and a dynamic task, in which the arm started at the side with 0 ° shoulder elevation, 0 ° elbow flexion, and the palm facing away from the midline at −45 ° shoulder rotation, shoulder elevation increased to 90 °, then returned to the start position with the arm at the side. To obtain the kinematics for both the static and dynamic tasks, 10 retro-reflective markers were placed on boney landmarks of a human size (plastic) skeleton on the left side of the skeleton. The skeleton was used to ensure proper placement on anatomical landmarks without soft tissue interference. Marker locations included: the radial and ulnar styloid, forearm, medial and lateral epicondyles, biceps, acromion process, head of clavicle, 7th cervical vertebra, and the xiphoid process. Briefly, 8 Kestrel cameras (Motion Analysis Corporation, Rohnert Park, CA) were used to track the location of the 10 markers. Marker locations were then post-processed and smoothed using a 6 Hz Butterworth filter using Cortex software (Motion Analysis Corporation). The filtered unilateral marker locations were then mirrored across the mid-sagittal plane to obtain the contralateral locations. Then both sides of marker locations were separately used as the input for all model simulations. For both verification tasks, the model wrist degrees of freedom (flexion/extension, pronation/supination) were locked at 0 °.

### Loading task simulations

**Fig 1:**
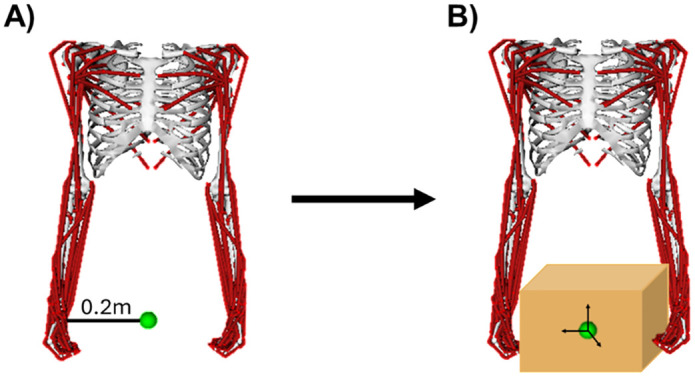
A) Default posture of the model for creating the shared load. The shared load center of mass was positioned halfway between the center of mass of the hands, 0.2 m from the left and the right. B) An image of a box was added to the model to better visualize the shared load.

Following verification, the model was then modified to include 1 of 2 different shared loads that represent regularly used objects (13.3 N (1.36 kg) and 44.5 N (4.5 kg)) at the hands. The loads were attached as a weld joint to the left hand, then attached to the right hand via a weld constraint [18]. The center of mass of the shared load was defined as the midpoint between the center of mass of the right and left hands, with palms facing toward the midline (thumbs up) and arms fully extended (Fig 1A). These loads were separately added in a posture that is representative of holding a box with both hands (Fig 1B). The models were then modified to represent 4 different unilateral RCT severities by reducing the peak isometric force of the supraspinatus, infraspinatus, and subscapularis muscle actuators in the model on the affected (model’s right) side, using our previously reported methods [19–21]. Using the assumption from prior work that muscle force is linearly related to the percentage of torn tendon, peak isometric muscle force for the injured muscles (supraspinatus, infraspinatus, and subscapularis) was reduced by a percentage of their nominal values within OpenSim. The RCT severities included: 1) no RCT – all muscles paths remained at 100% of baseline value; 2) a partial-thickness RCT – the supraspinatus muscle path was reduced to 50% of its baseline value; 3) a full-thickness RCT – supraspinatus was further reduced to 0% of its baseline value and infraspinatus was reduced to 75% of its baseline value; and 4) a massive RCT – infraspinatus was reduced to 25% of its baseline value and subscapularis muscle force was reduced to 50% of its baseline value.

**Fig 2:**
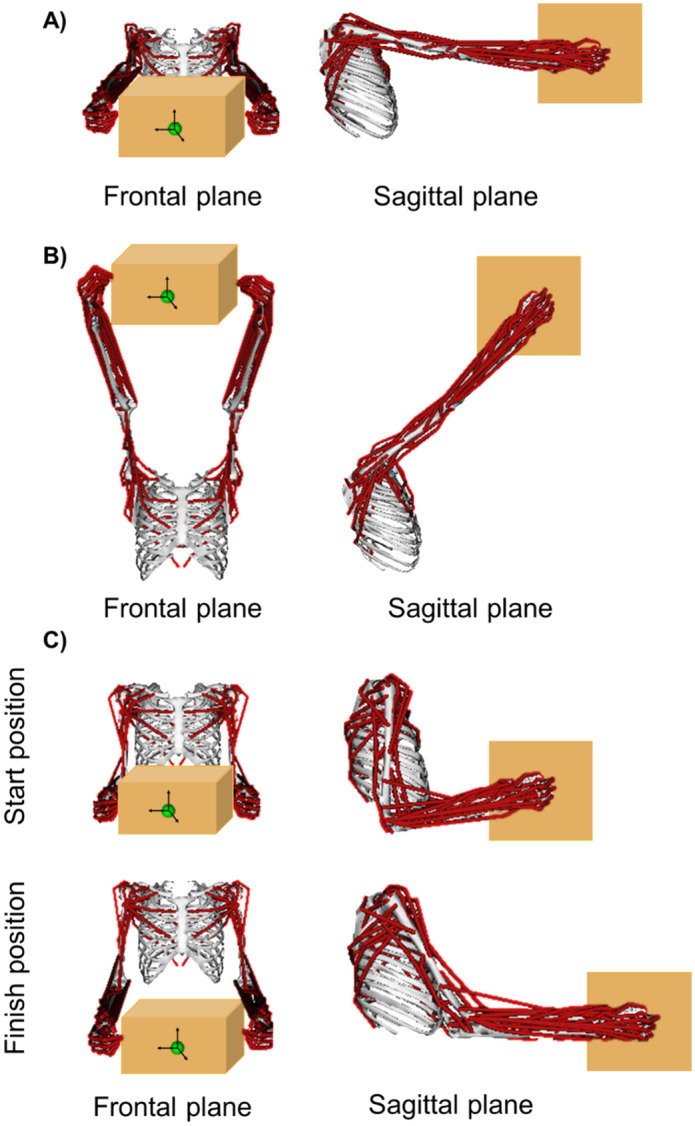
Frontal and sagittal views of the A) low posture static task: shoulder elevation was extended to 80 ° and elevation angle was set to 80° to replicate holding a box with the arms extended in front of the body at waist height; B) high posture static task: elevation angle remained at 80 °, and shoulder elevation was increased to 130 ° to represent holding a box with extended arms above eye height; C) dynamic task: the model started with the arms by the side at 90 ° elbow flexion, then reached forward in the sagittal plane until approximately 75 ° elevation angle and then returned to the start position.

Simulations were performed for the static and dynamic bimanual tasks. For the low posture static task, shoulder elevation was extended to 80 ° and elevation angle was set to 80 ° to replicate holding a box with the arms extended in front of the body at waist height (Fig 2A). For the high posture static task, elevation angle remained at 80 ° and shoulder elevation was increased to 130 ° to represent holding a box with extended arms above eye height (Fig 2B). Using data from a previous study [22], a randomly selected participant’s unilateral kinematics were used to define the bilateral joint kinematics for the dynamic task of a forward reach motion. The kinematic data used in this study was previously reported by Vidt et al. (2016) and was determined by the Pennsylvania State University Institutional Review Board (STUDY00012320) to be exempt from oversight [22]. Briefly, the kinematics consisted of the participant starting with their arm by their side at 90 ° elbow flexion. The participant then reached forward in the sagittal plane, then returned their arm to the start position. The joint angles were copied and mirrored across the mid-sagittal plane (in the global y-axis of the model) to obtain bilateral kinematics for the dynamic task (Fig 2C). Two static postures (low posture static task, high posture static task) and 1 dynamic task of a forward reach were used as separate inputs to the Computed Muscle Control (CMC) algorithm [23]. For the CMC setup, all kinematics were filtered at 6 Hz, the maximum step size was defined as 1, the minimum step size was defined as 0.0001, and the integrator error tolerance was set at 0.0005. CMC was used to predict muscle force for each task of the 13 muscle paths crossing the shoulder: anterior, middle, and posterior compartments of deltoid; infraspinatus; subscapularis; teres minor; teres major; clavicular, sternal, and ribs compartments of pectoralis major; and thoracic, lumbar, and iliac compartments of latissimus dorsi. Muscle force was then normalized to model-specific peak isometric force for further analysis. Following the CMC simulations, the CMC output of the simulation-predicted joint angles was used as the input to the point kinematics tool. The point kinematics algorithm was used to track the position of the box center of mass over the task duration for all model permutations.

### Outcomes and analysis

For model verification, Root Mean Squared Error (RMSE) was used to quantify the differences between the left- and right-side kinematics. For the loading simulation tasks, symmetry was not a criterion, instead the position of the box was quantified by calculating the amount the box deviated from the start position. For the static postures, maximum deviation from the starting position was determined using a custom MATLAB script (The MathWorks, Inc., Natick, MA). For the dynamic task, RMSE was calculated to quantify the difference in simulation-predicted box position relative to the position from the input kinematics using a custom MATLAB script. For the RMSE calculations, the input kinematics were defined as the target position and the simulation kinematics were the output. RMSE was used to define how much the simulation-predicted kinematics deviated from the input target kinematics in order to quantify the amount of deviation occurring during task performance. This data set only contains data from a single kinematic time history per task. Thus, traditional statistical analyses could not be performed; instead, quantitative trends were examined. The percentage change in average normalized muscle force (% maximum) between the model permutations was calculated. The maximum deviation of the box was determined for the inferior/superior and medial/lateral directions.

## Results

### Model verification

**Fig 3:**
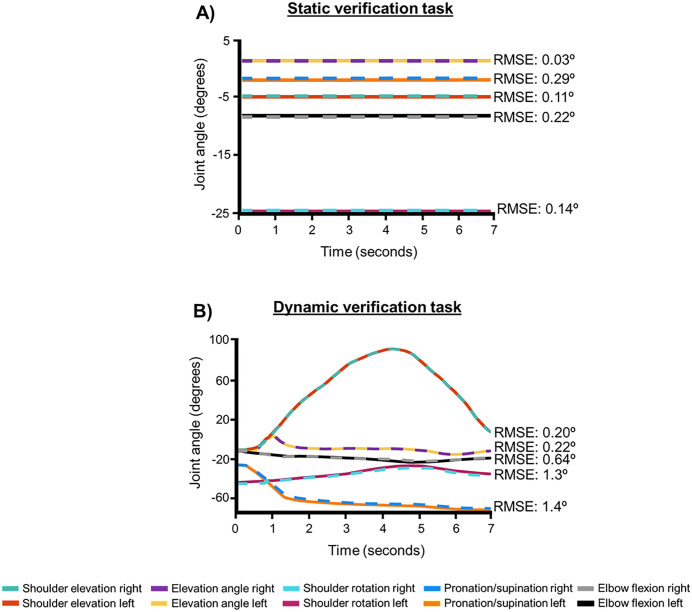
Left and right-side shoulder kinematics for model verification for: A) the static verification task, and B) the dynamic verification task. The left side is represented by the solid line and the right side is represented by the dashed line to show the overlap between the two sets of kinematics.

A bilateral model was further developed for assessment of bimanual tasks using computational simulations. Model kinematics were verified between right and left sides for prescribed static and dynamic tasks. Results showed that for the static verification task, minimal deviation occurred between the left and right sides for each degree of freedom: elevation angle, RMSE: 0.03 º; shoulder elevation, RMSE: 0.11 º; shoulder rotation, RMSE: 0.14 º; elbow flexion, RMSE: 0.22 º; and pronation/supination, RMSE: 0.29 º (Fig 3A). Similarly, for the single dynamic verification task, minimal deviation was observed between the right and left sides for each degree of freedom: elevation angle, RMSE: 0.22 º; shoulder elevation, RMSE: 0.20 º; shoulder rotation, RMSE: 1.3 º; elbow flexion, RMSE: 0.64 º; and pronation/supination, RMSE: 1.4 º (Fig 3B).

### Muscle force and shared load deviation during bimanual loading

**Fig 4:**
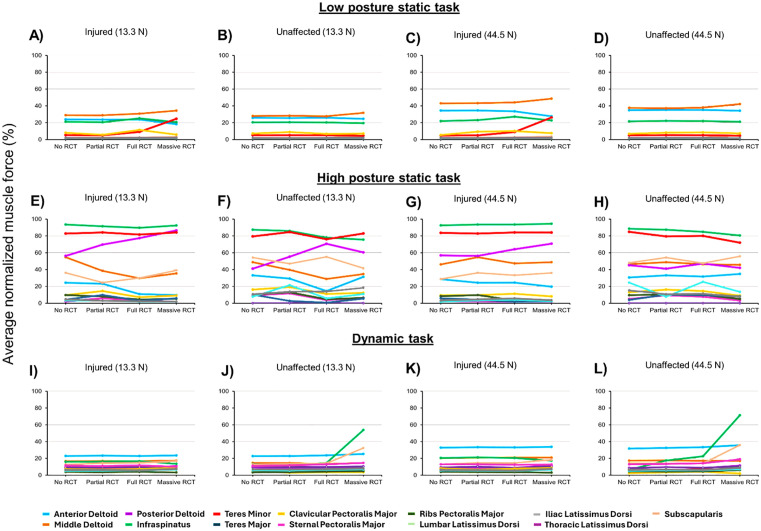
Low posture static task: Average muscle force contribution (% maximum) for each of the 13 muscle paths crossing the shoulder for the low posture static task for increasing RCT severity and external load for the A) injured side with the 13.3 N external load; B) unaffected side with the 13.3 N external load; C) injured side with the 44.5 N external load; D) unaffected side with the 13.3 N external load. **High posture static task:** Average muscle force contribution (% maximum) for each of the 13 muscle paths crossing the shoulder for the low posture static task for increasing RCT severity and external load for the **E)** injured side with the 13.3 N external load; **F)** unaffected side with the 13.3 N external load; **G)** injured side with the 44.5 N external load; **H)** unaffected side with the 13.3 N external load. **Dynamic task:** Average muscle force contribution (% maximum) for each of the 13 muscle paths crossing the shoulder for the low posture static task for increasing RCT severity and external load for the **I)** injured side with the 13.3 N external load; **J)** unaffected side with the 13.3 N external load; **K)** injured side with the 44.5 N external load; **L)** unaffected side with the 13.3 N external load.

**Fig 5:**
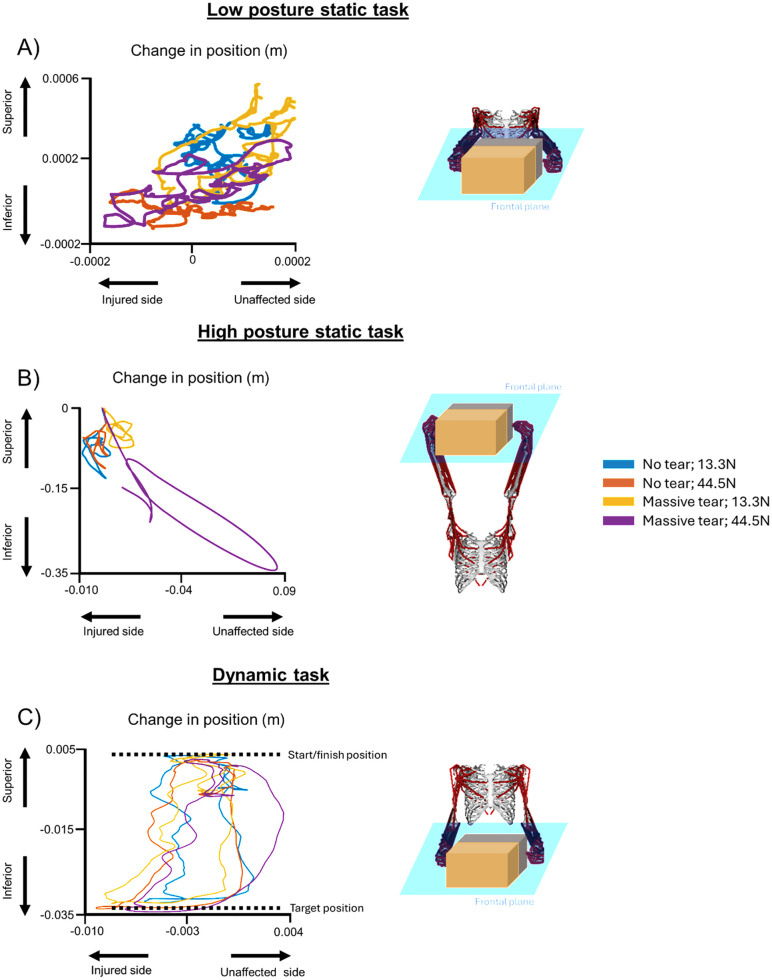
Change in position of the shared load center of mass across the no RCT and massive RCT models, each for: A) low posture static task; B) high posture static task; and C) dynamic task.

For the low posture static task**,** uninjured muscles on the injured side increased their average muscle force contribution (% maximum) to enable successful task performance with increased RCT severity and external load. For the low posture static task on the injured side, with loads of 13.3 N and 44.5 N applied, respectively, there was a 12% and 15% increase in average muscle force contribution (% maximum) for middle deltoid; 0% and 6% increase in average muscle force contribution (% maximum) for posterior deltoid; 35% and 44% increase in average muscle force contribution (% maximum) for subscapularis; and 79% and 82% increase for teres minor, between the no RCT and massive RCT severities (Fig 4A and 4C). For the low posture static task on the unaffected side, with loads of 13.3 N and 44.5 N applied, respectively, there was a 11% and 11% increase in average muscle force contribution (% maximum) for middle deltoid; 3% and 0% increase in average muscle force contribution (% maximum) for posterior deltoid; and 49% and 59% increase in average muscle force contribution (% maximum) for subscapularis, but for the unaffected side, there was a 13% and 9% decrease for teres minor, between the no RCT and massive RCT severities (Fig 4B and 4D). Minimal deviation was observed for all model permutations for the low posture static task, where maximum deviation was the greatest for the massive RCT with 44.5 N load condition, with 0.6 mm in the inferior direction and 0.2 mm toward the unaffected side (Fig 5A).

For the high posture static task, the deltoid, subscapularis, and teres minor increased force contribution, but were near maximum capacity for both the injured and unaffected sides. For the injured side, infraspinatus was near maximum capacity for the duration of the task, averaging 92% for the 13.3 N load and 93% for the 44.5 N load across all RCT severities. For the injured side, posterior deltoid increased average muscle force contribution (% maximum) by 35% for the 13.3 N but only 20% for the 44.5 N load, subscapularis increased average muscle force contribution (% maximum) by 7% for the 13.3 N load and 20% for the 44.5 N load, and teres minor had minimal force modulation with increasing RCT severity, with increases of 2% for the 13.3 N (Fig 4E) load and 0% for the 44.5 N load (Fig 4G). However, for the unaffected side, there were average muscle force contribution (% maximum) increases in the posterior deltoid of 32% for the 13.3 N load (Fig 4F) and in the anterior deltoid of 12% for the 44.5 N load (Fig 4H). Greater deviation occurred for the high posture static task, with the no RCT with 13.3 N load permutation, resulting in a maximum deviation of 149 mm in the inferior direction and 9 mm toward the unaffected side. For the massive RCT with the 44.5 N load permutation, deviation was 342 mm in the inferior direction and 83 mm toward the unaffected side (Fig 5B).

For the dynamic task, there were average muscle force contribution (% maximum) increases for muscles on the injured side, including the posterior deltoid by 25% for the 13.3 N load and 29% for the 44.5 N load, subscapularis by 24% for 13.3 N load and 30% for 44.5 N load, and teres minor by 24% for the 13.3 N load and 38% for the 44.5 N load from the no RCT to the massive RCT severity (Fig 4I and 4K). The unaffected side for the dynamic task showed average muscle force contribution (% maximum) increases in the posterior deltoid by 9% for the 13.3 N load and 24% for the 44.5 N load, subscapularis by 9% for the 13.3 N load and 61% for the 44.5 N load, and teres minor by 0% for the 13.3 N load and 20% for the 44.5 N load from the no RCT to the massive RCT severity (Fig 4J and 4L). However, during the dynamic task, there were average muscle force contribution (% maximum) decreases in the infraspinatus muscle on the injured side by 18% for the 13.3 N load and 19% for the 44.5 N load from the no RCT to the massive RCT severity (Fig 4I and 4K), while there were large average muscle force contribution (% maximum) increases for infraspinatus on the unaffected side by 76% for the 13.3 N load and 91% for the 44.5 N load (Fig 4J and 4L). Deviation of the box from the input kinematics was minimal for the dynamic task. For the dynamic loading task, with the no RCT model with 13.3 N load, RMSE was 6.7 mm in the inferior direction and 2.1 mm toward the unaffected side. Similar trends were observed in box deviation for the dynamic task for the massive RCT, 44.5 N load, where RMSE was 7.2 mm in the inferior direction and 1.5 mm toward the unaffected side (Fig 5C).

## Discussion

This study sought to further develop a unilateral upper extremity musculoskeletal model to a bilateral model to examine differences in bilateral muscle compensation strategies and changes in kinematics while performing a loaded bimanual task with a unilateral RCT. This work found that uninjured muscles on the injured side increased their average muscle force contribution (% maximum) with increased RCT severity for all tasks. This is consistent with what was expected. However, bilateral muscle compensation was expected to occur in all tasks but was only observed for the dynamic task with the massive RCT severity. For the low posture static task, minimal bilateral force modulation and kinematic deviation occurred for any permutation of RCT severity and external load. For the high posture static task, forces were near maximum capacity for both the injured and unaffected sides. This lack of muscle compensation resulted in increased deviation inferiorly and toward the uninjured side.

The current unilateral musculoskeletal model was further developed to create a bilateral model. This newly developed model was verified using a static and dynamic kinematic trajectory. While a bilateral model has been previously published [15], the kinematic verification of that model was not reported. The model developed here resulted in minimal (all degrees of freedom <1.5 º) differences between the left and right side kinematics for both static and dynamic verification tasks. The same verification tests were also performed on the previously published bilateral model, and it was found that RMSE values from simulations performed using the prior model were greater between the right and left side kinematics (both tasks, all degrees of freedom <6.0º) than the RMSE values computed from simulations performed using the model developed in the current study. In addition to the analyses discussed here, future work should include additional sensitivity analyses in conjunction with experimental work to confirm the results provided here. Due to the smaller differences between right and left side shoulder degrees of freedom for the current implementation of the bilateral model than the previously published model, the model developed in this study was used to perform the current assessment of bilateral muscle compensation for unilateral RCT. RMSE values were expected to be minimal, but not zero, which would account for rounding differences that occurred during data processing to mirror the unilateral kinematic data prior to running simulations. Specifically, the raw kinematic data was mirrored by multiplying the x-coordinate for all marker locations by −1 to reflect the coordinate about the x-axis. It is possible that the addition of the negative sign in the software could have caused small rounding differences than when the negative sign was not present in the original kinematic data.

Using the verified model developed for this study, simulations of static tasks at both low and high postures resulted in minimal muscle compensation on the unaffected side, suggesting that bilateral compensation is not occurring during performance of the loaded static tasks. Other simulation results showed that muscles, such as the deltoid and teres minor on the unaffected side, compensated with increased RCT severity and external load during a dynamic task. This is consistent with prior experimental studies that showed increased compensation from the deltoid and teres minor in the context of RCT [10,11]. The current study’s results for the low posture static task and for the high posture static task do not support the hypothesis that muscles on the unaffected side would increase force contribution in the context of increased RCT severity. For the dynamic task, the results confirmed the hypothesis that muscles on the unaffected side would increase their force contribution in the context of increased RCT severity. It is important to note that although bilateral compensation did not occur for the low posture and high posture static tasks, the duration of the task (7 s) might be affecting the results. Future work should consider examining different durations of task performance to better understand how the length of task affects muscle compensation, which will provide insight into how long duration postures should be maintained before muscles are at maximum capacity.

This work observed muscle force contributions (% maximum) that were nearing maximum capacity during the high posture static task. This is not consistent with prior literature that observed muscle activity at approximately 20% of the maximum voluntary contraction [24]. This discrepancy in simulation-predicted results and experimental results could be due to postural differences that occurred. The defined posture for the model consisted of fully flexed arms, which may not directly reflect *in vivo* behavior. Future use of the model should include additional analyses that consider the effects of postural dependency, with further analyses on how muscle compensation changes with slight changes to posture. The dynamic task required increases in muscle force contributions (% maximum) from the unaffected side to enable task completion. During the dynamic task, muscle contribution ranged from 20–40% of maximum capacity. This is consistent with literature that resulted in muscle activation of ~ 40% during a dynamic tracking task [25]. However, little work has examined the effect of RCT on bilateral muscle compensation during a dynamic task, despite a majority of daily tasks being bimanual in nature. The results shown here suggest that during dynamic task performance, muscles on the unaffected side are compensating to improve task performance. Outcomes of this work suggest that muscles on the unaffected side are good targets for strengthening in addition to the current rehabilitation methods that often focus on strengthening muscles on the injured side [26]. In addition to bilateral muscle compensation occurring for the dynamic task, this work found that unilateral compensation also occurred to enable task performance. The results from all tasks confirmed our hypothesis that uninjured muscles on the injured side would increase force contribution in the context of increased RCT severity. Muscle force contributions (% maximum) increased in uninjured muscles (deltoid and teres minor) on the injured side for the low and high posture static tasks and the dynamic task, suggesting that unilateral muscle compensation occurred on the injured side. This is consistent with previous computational and experimental work that found force increases on the injured side for the deltoid and teres minor muscles following RCT injury [10–12]. However, more work should be performed to further understand how individual muscles and muscle groups compensate for a unilateral RCT during bimanual task performance.

Kinematic deviation was minimal for the low posture static task and the dynamic task, suggesting successful task completion. The minimal deviations observed in kinematic deviation for the low posture static task are likely not clinically significant. However, future work is needed to define thresholds of deviation that are clinically meaningful. Our findings showed decreased deviation of the box across all tasks for the low load compared to the high load. The minimal deviation observed in this study suggests that the model is able to maintain the position of the shared load. At high loads, greater deviation of the box occurred, where deviation was increased in the inferior direction compared to the low posture static task and dynamic task. This was likely because for the high posture static task, muscle forces were near maximum capacity for all RCT severities, which would decrease the ability of muscle compensation to occur. Due to the high force contributions and increased deviation, the results indicated that with increased injury, statically maintaining a load above eye height resulted in deviation of the box. The deviation at the high loads observed in the current study could contribute to further progression of the RCT or development of a secondary injury [27]. It is important to note here that the developed model represents the shoulder as a pure ball-and-socket joint. Further development of the model to account for humeral head translation [28] and independent scapular motion would allow for more in-depth analyses for what could be expected for kinematic deviation while performing a loaded task with a unilateral RCT.

The occurrence of kinematic deviation while moving an external load is consistent with previous work [29]. In the current study we observed deviation of the box toward the uninjured side of the model, suggesting that a greater proportion of the shared load is borne by the uninjured side despite there not being any muscle force contribution (% maximum) increases on the unaffected side. The increased deviation in the high posture static task could result in difficulty completing overhead working tasks, particularly those that are prolonged. During the dynamic task, deviation from the input kinematics was minimal across all model permutations, but there was an increase in force contribution from muscles on the unaffected side. The minimal deviation observed for the dynamic task could be due to the increase in force contribution from the unaffected side, suggesting that bilateral muscle compensation enables successful task performance. The work presented here is consistent with other work by our group that showed unilateral kinematic declines and muscle compensation decreases in planar versus multi-planar tasks when moving an external load with a RCT.

This work first aimed to develop a bilateral musculoskeletal model, validate the model, and then use it to examine differences in task performance. The developed model did produce low error when comparing between the left and right sides, however, further validation of the model is warranted for future use. This is a limitation of the current work, as the model needs to be further validated to ensure proper mirroring of the data and for studies involving specific clinical populations. Despite this limitation, this work provides the model to other researchers for further use and similar validation. Other limitations of this work include only having a single representative kinematic trajectory for each task, as the postures were defined for the simulations. This did not permit traditional statistical analyses, which are beneficial to fully understanding the observed trends. However, this work provided a bilateral model that can be used in future studies to further examine these trends statistically. As only a single kinematic trajectory was evaluated for each task, the current results do not account for postural variation across individuals that could impact these results [30]. The modeling framework developed here enables further studies into the influence of postural variability. The CMC simulations to determine muscle force have not yet been validated in the bilateral model, but they have been validated for the unilateral model. Additional validation of CMC for the new bilateral model developed here will be beneficial for future use of the model. The muscle force results found in this study are consistent with prior work that showed muscle force increases in injured muscles on the affected side using both experimental and computational methods [10–12]. Also, work did not assess changes in joint contact forces (JCFs). Prior work states that JCFs change in the context of unilateral RCT severity [20], but it is unknown how these trends are different for bilateral JCFs in the context of RCT. Future work should include JCF analyses to better understand the effect of injury on bilateral compensation. The current study only examined the average muscle force (% maximum) for each task. Further examination of continuous muscle force during task performance may provide deeper insight into how and when during task performance muscles alter their force contribution to compensate. The different RCT severities were modeled by decreasing the peak isometric force of the injured tendons; this method does not account for other biological changes that may occur following RCT, such as muscle atrophy, altered kinematics to account for pain, muscle stiffness, or neural differences. However, this method of modeling RCT has been established in prior work [19] and is consistent with prior literature using both computational and experimental methods [12,19]. In addition, the current results provide information on the relative changes that could be expected in muscle compensation strategies following a unilateral RCT when performing bimanual tasks. The method of adding a shared load to the hands has been validated for spine loading [16], not loading at the shoulder. As the authors are unaware of prior work, this study presents a method to study the effects of a shared load at the hands in the context of shoulder loading. Due to this, future work should perform experiments to validate the method of adding a shared load to the hands. Despite the limitations, this model and the results can still provide insight into the types of changes we can expect to occur in the context of RCT.

Overall, this study further developed an existing unilateral model to create a bilateral upper extremity model and used computational simulations to show that bilateral compensation occurs to enable successful task performance. This work also found that performance of a loaded overhead static task with a RCT results in greater kinematic deviation compared to a low static posture. This work highlights that bilateral compensation may be an important aspect to assess during treatment and rehabilitation, specifically for individuals who work in occupations that require overhead work or working in awkward positions. The results from this study identified that the deltoid, infraspinatus, and subscapularis muscles are compensating both on the injured side and unaffected side in the context of RCT. These findings could be used in the development of rehabilitation strategies that target specific muscles for strengthening. However, we urge caution when extrapolating the results from this work to immediate design of rehabilitation because the outcomes of this study need to be further validated using experimental methods or additional analyses. The current study found that muscles compensate for a unilateral RCT during bimanual task performance. Additional studies should be performed to include experimental measures, such as electromyography (EMG) or strength testing, to obtain an improved understanding of the trends observed here. The bilateral model developed in this study can be used to expand on the trends observed here by examining the effects of RCT and load on the elbow and wrist joints. Additionally, the model can be used to examine task performance in the context of bimanual tasks, such as bimanual compensation, the effects of handedness during task performance, and the effects of injury on bimanual task performance, all of which are difficult to examine with a unilateral model. The identification of the compensatory mechanisms of unilateral injury has the potential to expose targets for customized rehabilitation to enable performance of daily functional and occupational tasks, and permit further studies of the mechanisms driving development and progression of musculoskeletal disorders, like RCT.

## Supporting information

S1 File MUSL_Unscaled_Bilateral_June24.osim: The newly developed bilateral musculoskeletal model. (OSIM)

S2 File External_force_13_3N.mot: The 13.3N external force used during model simulations. (MOT)

S3 File HighPostureStaticTask.mot: The high posture static task used as the input for model simulations. (MOT)

S4 File LowPostureStaticTask.mot: The low posture static task used as the input for model simulations. (MOT)

## Abbreviations

CMC: Computed muscle control
RCT: Rotator cuff tear
RMSE: Root mean square error.
